# The Contribution of Tissue Inhibitor of Metalloproteinase-2 Genotypes to Breast Cancer Risk in Taiwan

**DOI:** 10.3390/life14010009

**Published:** 2023-12-20

**Authors:** Yun-Chi Wang, Jie-Long He, Chung-Lin Tsai, Huey-En Tzeng, Wen-Shin Chang, Shih-Han Pan, Li-Hsiou Chen, Chen-Hsien Su, Jiunn-Cherng Lin, Chih-Chiang Hung, Da-Tian Bau, Chia-Wen Tsai

**Affiliations:** 1 Graduate Institute of Biomedical Sciences, China Medical University, Taichung 404333, Taiwan; 2 Terry Fox Cancer Research Laboratory, Department of Medical Research, China Medical University Hospital, Taichung 404327, Taiwan; 3 Department of Post-Baccalaureate Veterinary Medicine, Asia University, Taichung 413305, Taiwan; 4 Division of Cardiac and Vascular Surgery, Cardiovascular Center, Taichung Veterans General Hospital, Taichung 407219, Taiwan; 5 Division of Hematology/Medical Oncology, Department of Medicine, Taichung Veterans General Hospital, Taichung 407219, Taiwan; 6 Ph.D. Program for Cancer Molecular Biology and Drug Discovery, and Graduate Institute of Cancer Biology and Drug Discovery, College of Medical Science and Technology, Taipei Medical University, Taipei 110301, Taiwan; 7 Division of Cardiology, Department of Internal Medicine, Taichung Veterans General Hospital, Chiayi Branch, Chiayi 60090, Taiwan; 8 Division of Breast Surgery, Department of Surgery, Taichung Veterans General Hospital, Taichung 407219, Taiwan; 9 Department of Bioinformatics and Medical Engineering, Asia University, Taichung 413305, Taiwan

**Keywords:** breast cancer, genotype, polymorphism, tissue inhibitor of metalloproteinase-2

## Abstract

Tissue inhibitor of metalloproteinase-2 (TIMP-2) is an endogenous inhibitor of matrix metalloproteinase-2 and is highly expressed in breast cancer (BC) cases at diagnosis. However, the genetic investigations for the association of *TIMP-2* genotypes with BC risk are rather limited. In this study, contribution of *TIMP-2* rs8179090, rs4789936, rs2009196 and rs7342880 genotypes to BC risk was examined among Taiwan’s BC population. *TIMP-2* genotypic profiles were revealed among 1232 BC cases and 1232 controls about their contribution to BC using a PCR-based RFLP methodology. The *TIMP-2* rs8179090 homozygous variant CC genotype was significantly higher in BC cases than controls (odds ratio (OR) = 2.76, 95% confidence interval (95%CI) = 1.78–4.28, *p* = 0.0001). Allelic analysis showed that C allele carriers have increased risk for BC (OR = 1.39, 95%CI = 1.20–1.62, *p* = 0.0001). Genotypic together with allelic analysis showed that *TIMP-2* rs4789936, rs2009196 or rs7342880 were not associated with BC risk. Stratification analysis showed that *TIMP-2* rs8179090 genotypes were significantly associated with BC risk among younger (≤55) aged women, not among those of an elder (>55) age. Last, rs8179090 genotypes were also associated with triple negative BC. This study sheds light into the etiology of BC in Taiwanese women. Rs8179090 may be incorporated into polygenic risk scores and risk prediction models, which could aid in stratifying individuals for targeted breast cancer screening.

## 1. Introduction

Breast cancer (BC) stands as the foremost malignancy among women globally [1,2]. Among its various subtypes, triple-negative breast cancer (TNBC) constitutes 15–20% of BC cases [3]. Clinically, TNBC demonstrates a high aggressiveness, with the capacity for metastasis to diverse organs such as the brain, lungs, heart, liver, and bones [4]. Notably, the 5-year survival rates for TNBC patients are notably lower compared to other BC subtypes (77% versus 93%) [5]. In Taiwan, BC has consistently held the highest incidence among cancers [6,7]. Moreover, BC in Taiwan is characterized by a marked surge in prevalence and relatively early onset (diagnosis at 45–49 years) [6,8]. Our findings provide evidence suggesting that *MTHFR* genotypes might contribute to BC susceptibility and potentially play a role in the early onset of BC in Taiwan, aligning with alternative hypotheses proposed by other research groups [9,10,11,12]. Currently, one of the focal points for translational scientists is the search for practical biomarkers for BC, particularly TNBC in Taiwan [13,14,15].

Tissue inhibitor of metalloproteinase-2 (TIMP-2) plays a critical role in regulating tumor invasion by modulating the activity of MMP-2 [16]. A study dating back to 2002 by Nakopoulou and his colleagues examined 136 BC samples, revealing that larger tumor sizes in BC patients were associated with negative TIMP-2 expression [17]. Conversely, elevated TIMP-2 expression was often observed in low-grade BC cases, correlating with a better survival rate compared to those exhibiting normal TIMP-2 expression [17]. A 2020 study by Peeney and his colleagues reported that TIMP-2 could suppress the growth and metastasis of TNBC cells by modulating the epithelial-to-mesenchymal transition and signaling pathways associated with metastatic outgrowth [18]. Subsequently, a 2022 study found significantly higher TIMP-2 expression among 96 BC patients compared to 35 healthy individuals [19]. However, the precise contribution of elevated TIMP-2 to BC etiology remains unclear. On the other side, genetic variations within the *TIMP-2* gene, located on chromosome 17q25, may potentially alter its activity, disrupting the balance between TIMP-2 and MMP-2 activity. This disrupted equilibrium could significantly impact the development and progression of tumor cells [20]. Accumulated research indicated that *TIMP-2* genetic variants may influence the risk of various types of cancer, including head and neck cancer, prostate cancer, and gastric cancer [21,22,23]. Collectively, these findings suggested that assessing *TIMP-2* polymorphisms, in addition to its expression levels, could serve as a valuable biomarker for various types of cancer.

**Figure 1:**
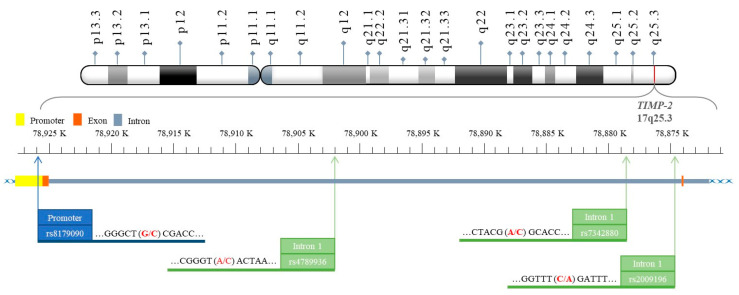
Physical map of *TIMP-2* rs8179090, rs4789936, rs2009196 and rs7342880 polymorphic sites.

Only a handful of studies worldwide have investigated the single nucleotide polymorphisms (SNPs) of *TIMP-2* in individuals with BC [24,25,26,27]. In this study, we used a hospital-based case control study to compare the genotype frequencies of four SNPs in *TIMP-2* gene, rs8179090, rs4789936, rs2009196, and rs7342880, between BC patients and healthy controls and determine the associations between these SNPs and BC risks. Additionally, we aim to provide evidence of the role of *TIMP-2* genotypes in predicting TNBC risk. The physical map for the investigated SNPs in this study is shown in Figure 1. 

## 2. Materials and Methods

### 2.1. BC and Non-BC Control Population

A total of 1232 patients diagnosed with BC were enrolled from the outpatient clinics of the general surgery department at China Medical University Hospital in Taiwan for this study. All participants were of Taiwanese descent, and the detailed procedures, exclusion and inclusion criteria were previously documented [28,29,30]. Clinical characteristics, including histological details, were defined by Dr. Su and his team. BC tissue slides were independently reviewed and scored by at least two pathologists. Positivity for ER, PR, and HER-2/neu immunoassaying was determined via nuclear staining in 10% of neoplastic cells. A Ki67-labeling index of >30% was considered positive. HER-2/neu results adhered to the guidelines of the American Society of Clinical Oncology and the College of American Pathologists [31]. All patients voluntarily participated, completed a self-administered questionnaire, and provided peripheral blood samples. As controls for this study, 1232 age-matched healthy volunteers were selected through initial random sampling from the health examination cohort of the hospital. Exclusion criteria for the control group included previous malignancies, metastasized cancer of other or unknown origins, and any familial or genetic diseases. Both groups completed a brief questionnaire that included lifestyle habits. Our study received approval from the Institutional Review Board of China Medical University Hospital (DMR-96-IRB-240), and written informed consent was obtained from all participants.

### 2.2. TIMP-2 Genotyping Methodology

Peripheral blood was collected from all participants, and their DNA was extracted within 24 h and stored according to our routine protocol [32,33]. Genotyping for *TIMP-2* rs8179090, rs4789936, rs2009196, and rs7342880 was performed using the polymerase chain reaction restriction fragment length polymorphism (PCR-RFLP) methodology as we previously published [34]. Briefly, the primer sequences for *TIMP-2* genotyping were designed and optimized by the Terry Fox Cancer Research Lab. The forward and reverse primer sequences for *TIMP-2* rs8179090 were TTCTAAGGCCTCCATTTGAA and GTTCTTCCAGGACACCAGGC, respectively. For *TIMP-2* rs4789936, the forward and reverse primer sequences were CCATCTACAGAGATGCCAGT and TAAGCTGAGATCGCACCACT, respectively. The forward and reverse primer sequences for *TIMP-2* rs2009196 were GACTGAAGCTCATCTGTTGA and CGCGAGACTCCATCTCAATA, and for *TIMP-2* rs7342880, the sequences were GCCACAGTTGTTCACACCTA and GGACCCTGAAGAATCTGAAT. PCR was conducted using a PCR thermocycler (Bio-RAD, Hercules, CA, USA) under the following conditions, initial denaturation at 94 °C for 5 min, followed by denaturation at 94 °C for 30 s, annealing at 64 °C for 40 s, and extension at 72 °C for 45 s. Last, after 35 PCR cycles, a final extension was performed at 72 °C for 10 min. The PCR products were analyzed using 3% agarose gel electrophoresis. For *TIMP-2* rs8179090, the G allele was digested by *Mnl* I, resulting in two fragments of 117 and 119 base pairs (bps), while the undigested T allele remained at 236 bps. Similarly, for *TIMP-2* rs4789936, the C allele was digested by *BtsIMut* I, yielding two fragments of 179 and 292 bps, while the undigested A allele remained at 471 bps. For *TIMP-2* rs2009196, the C allele was digested by *Mwo* I, resulting in two fragments of 116 and 251 bps, while the undigested G allele remained at 367 bps. For *TIMP-2* rs7342880, the A allele was digested by *BssS* I, resulting in two fragments of 135 and 418 bps, while the undigested C allele remained at 553 bps. We sent ten DNA samples with representative genotypes and the results of PCR-RFLP and sequencing were 100% concordant. Genotyping was independently and blindly repeated by two researchers, and all procedures yielded consistent results with 100% concordance. The genotype frequencies of all the four SNPs were in the Hardy–Weinberg equilibrium. 

### 2.3. Analyzing Methodology

The age distribution difference between the case and control groups was evaluated using the typical Student’s *t*-test. Pearson’s chi-square test was employed to assess the differential distribution of the *TIMP-2* genotypes. The associations between the *TIMP-2* genotypes and BC risk were analyzed using multivariable logistic regression. Odds ratios (ORs) and corresponding 95% confidence intervals (CIs) were calculated in overall population and in various stratification analyses. To increase statistical power, we recruited as many cases and controls as possible. Our sample size of 1232 pairs of BC patients and healthy controls gives us 90% power to detect an OR of 1.37 for a SNP like rs8179090 with a risk allele frequency of 18.9%. A *p*-value below 0.05 was deemed statistically significant for all outcomes.

## 3. Results

### 3.1. The Demographic Comparisons of the Taiwan BC Population

The comparison of age, age at menarche, age at first childbirth, age at menopause, personal habits, family history, tumor sites, and TNBC status between the 1232 BC cases and 1232 controls is presented in Table 1. Initially, no significant differences were observed between the case and control groups in terms of age, age at menarche, age at first childbirth, and age at menopause (all *p* > 0.05) (Table 1). Secondly, the prevalence of smokers and alcohol consumers was notably higher in the BC patient group than in the control group (both *p* < 0.0001) (Table 1). Lastly, among the 1232 BC cases, 194 were identified as TNBC cases, and the majority (97.2%) of BC cases were unilateral (Table 1).

### 3.2. The Genotypic Patterns of TIMP-2 in Taiwan BC Population

The genotypic distributions of *TIMP-2* among the 1232 controls and 1232 BC cases are presented in Table 2. Initially, the frequencies of *TIMP-2* rs8179090, rs4789936, rs2009196, and rs7342880 genotypes among the controls all conformed to the Hardy–Weinberg equilibrium (*p* = 0.3886, 0.1735, 0.3904, and 0.8422). Subsequently, significant differences were observed in the distribution of *TIMP-2* rs8179090 genotypes between the BC and control groups (*p* for trend = 1.00 × 10^−5^). In detail, carriers of the *TIMP-2* rs8179090 heterozygous variant CG and homozygous variant CC genotypes exhibited 1.16- and 2.76-fold increased ORs for BC risk, respectively, compared to individuals with the wild-type GG genotype (95%CI = 0.97–1.39 and 1.78–4.28, *p* = 0.1249 and 0.0001, respectively). The latter association was considered statistically significant. Furthermore, individuals with the homozygous variant CC genotype showed a significantly higher risk for BC than those carrying GG + CG genotypes in the recessive model (OR = 2.65, 95%CI = 1.71–4.10, *p* = 0.0001). Additionally, carriers of the *TIMP-2* rs8179090 CG + CC genotypes exhibited a significantly higher risk for BC than GG carriers in the dominant model (OR = 1.30, 95%CI = 1.09–1.55, *p* = 0.0034). Notably, carriers of variant CG and CC genotypes at *TIMP-2* rs8179090 displayed a decreased risk for BC. Regarding *TIMP-2* rs4789936, rs2009196, and rs7342880, no variant genotype was found to be associated with altered BC risk in any of the examined models (Table 3, Table 4 and Table 5 for each SNP).

### 3.3. The Patterns of TIMP-2 Allelic Frequencies in Taiwan BC Population

The allelic frequency distribution analyses for the four *TIMP-2* SNPs were performed to confirm the findings presented in Table 2, Table 3, Table 4 and Table 5. Consistently, the sole significant observation was that the variant C allele of *TIMP-2* rs8179090 exhibited a higher frequency (18.9%) in the BC group compared to the control group (14.3%) (OR = 1.39, 95%CI = 1.20–1.62, *p* = 0.0001, as shown in Table 6).

### 3.4. TIMP-2 Rs8179090 Genotypes Correlated with Onset Ages in Determining BC Risk

The genotyping results for *TIMP-2* rs8179090 were further stratified by age among both cases and controls (as presented in Table 7) to investigate the interaction between *TIMP-2* rs8179090 genotype and age concerning BC risk. Intriguingly, the homozygous variant CC genotype at *TIMP-2* rs8179090 showed an increased association with BC risk among individuals aged 55 years or younger (OR = 3.67, 95%CI = 2.11–6.38, *p* = 0.0001). Conversely, the CC genotypes at *TIMP-2* rs8179090 did not exhibit an altered BC risk among individuals aged over 55 years (OR = 1.47, 95%CI = 0.69–3.14, *p* = 0.4236) (Table 7).

### 3.5. TIMP-2 Rs8179090 Genotypes Were Associated with TNBC Risk

We sought to investigate whether the *TIMP-2* rs8179090 genotype could serve as a biomarker for predicting TNBC risk. Therefore, the BC patients were further stratified into TNBC and non-TNBC subgroups. The findings revealed significant associations between the *TIMP-2* rs8179090 homozygous variant CC genotype and both BC and TNBC. Among both TNBC and non-TNBC cases, the presence of the *TIMP-2* rs8179090 homozygous variant CC genotype showed significant associations with BC and TNBC (OR = 2.63 and 3.48, 95%CI = 1.67–4.14 and 1.79–6.76, *p* = 0.0001 and 0.0003) (Table 8).

## 4. Discussion

The major finding of this study is that the TIMP-2 SNP rs8179090 homozygous variant CC genotype was associated with a 2.76-fold increased risk, and the C allele associated with a 1.39-fold increased risk of breast cancer in Taiwanese women. The other three SNPs, rs4789936, rs2009196 or rs7342880, were not associated with BC risk. Moreover, rs8179090 genotypes can also serve as a biomarker for triple negative BC. 

Consistent with our results, a previous study evaluated 19 *TIMP-2* SNPs in a Chinese population consisting of 1062 BC cases and 1069 healthy controls and found that rs8179090 variant genotypes were significantly associated with BC risk. They also found that another nearby SNP, rs7501477, exhibited a 3-fold higher likelihood of BC with the TT variant genotype compared to women with the wild-type CC genotype [26]. We were the first to examine the contribution of *TIMP-2* genotypes to the risk of BC in Taiwan. Moreover, we found that the genotypes of rs8179090 can serve as predictors for the occurrence of TNBC (Table 8). Furthermore, we discovered that the genotypes of rs8179090 were selectively associated with BC susceptibility among women younger than 55 years old (Table 7). Consequently, we propose applying this novel marker for early detection of BC risk in young women. 

There were some reports examining the expression level of TIMP-2. In 2019, Wang and his colleagues reported significantly elevated transcriptional expression levels of TIMP-2 among tumor sites from 1097 BC cases compared to 114 normal controls, which conflicts with, yet holds greater persuasiveness in terms of sample size, than the findings presented by Ozdemir, which involved only 96 BC patients and 35 healthy individuals [19]. Furthermore, in 2022, Krasnikova and co-authors observed a notable decrease in serum TIMP-2 levels post-BC chemo-treatments among 67 BC patients compared with 25 healthy subjects [35]. The authors suggested that reduced serum TIMP-2 could potentially serve as an indicator for endothelial dysfunction resulting from anti-tumor therapy. Nakopoulou [17] and Jones [36] observed comparable immuno-activity of TIMP-2 in breast primary cancer cells and fibroblasts, while Garbett and colleagues reported heightened TIMP-2 expression in tumor cells compared to fibroblasts and inflammatory cells [36]. Nevertheless, these studies were constrained by small sample sizes and varied types of chemo-treatments. In 2020, Peeney and his colleagues provided evidence from an orthotopic mice model demonstrating the effective suppression of growth and metastasis of TNBC cells by TIMP-2 [18]. 

In the current study, for rs4789936, rs2009196, and rs7342880 we did not find significant associations (Table 3, Table 4 and Table 5), while rs8179090 can serve as a novel marker for BC risk (Table 2). In the literature, there were several reports revealing the genotypes of *TIMP-2* and then evaluated their contributions to BC. In 2009, the BC study by Peterson and colleagues evaluated the contribution of *TIMP-2* genotypes to BC risk using 19 *TIMP-2* polymorphic sites in a population consisting of 1062 BC cases and 1069 healthy controls. Their data supported our finding that promoter polymorphic rs8179090 genotypes were significantly associated with BC risk (Table 2 and Table 6). They also found that a polymorphic site nearby, rs7501477, exhibited a 3-fold higher likelihood of BC cases with the TT variant genotype compared to women with the wild-type CC genotype [26]. The samples collected in our current study are both genetically and geographically consistent and notably larger. Additionally, we were the first to examine the contribution of *TIMP-2* genotypes to the risk of TNBC, revealing that the genotypes of *TIMP-2* rs8179090 can serve as predictors for the occurrence of TNBC (Table 8). Furthermore, we discovered that the genotypes of *TIMP-2* rs8179090 were selectively associated with BC susceptibility among women younger than 55 years old (Table 7). Liu and his colleagues reported that the variant genotypes of the rs4789936 were associated with an increased risk of BC in a study of 480 Chinese BC patients and 530 healthy controls [25]. Wang and his colleagues investigated four SNPs, including rs2277698, rs2009196, rs7342880, and rs4789936 in another Chinese population comprising 566 BC patients and 578 healthy controls. Consistent with our results, they did not find significant associations for the rs2009196 and rs7342880 SNPs.

TIMP-2 is an endogenous inhibitor of matrix metalloproteinases that are involved in cancer development and progression. There is ample literature supporting that TIMP-2 exhibits antitumor activities, inhibiting tumor cell growth, angiogenesis, epithelial-mesenchymal transition (EMT), and metastasis [18,37,38]. TIMP-2 is downregulated or silenced in a variety of cancers [39,40]. For example, TIMP-2 is hypermethylated and silenced in prostate cancer cell lines and primary tumors, and silenced *TIMP-2* gene expression is associated with cancer progression during the invasive and metastatic stages of the disease. Furthermore, re-expression of TIMP-2 in metastatic prostate cancer cells significantly inhibited cell invasion [40]. Nakopoulou et al. found that increased tumor volumes often correlated with negative TIMP-2 expression [17]. Additionally, higher TIMP-2 levels were detected in most cases of low-grade BC patients, correlating with longer survival periods [17]. Furthermore, Krasnikova et al. observed a notable decrease in serum TIMP-2 levels post-BC chemo-treatments [35]. Notably, Peeney and colleagues demonstrated in an orthotopic mouse model that TIMP-2 can suppress the proliferation and metastasis of TNBC tumor cells [18]. All these data support a tumor-suppressive role of TIMP-2 in BC.

Rs8179090 is located at position −418 in the consensus sequence for the Sp1 binding site within the core promoter region of the *TIMP-2* gene [41]. Sp1 protein binds to the consensus sequence and stimulates transcriptional activity. Therefore, it is hypothesized that a G/C transition at −418 position results in downregulation of the transcriptional activity of the promoter, leading to reduced TIMP-2 expression. Reduced TIMP-2 would increase BC risk. It is therefore biologically plausible for the significant associations between the CC genotype and C allele and increased BC risk.

Giving that TIMP-2 serves as an endogenous inhibitor of MMPs, the precise reason for how variant genotypes may influence individual susceptibility to BC remains unclear. Multiple studies have suggested that, alongside its inhibitory effects on MMP-2/9, TIMP-2 can foster tumor cell proliferation, facilitate invasiveness/metastasis, and impede tumor cell apoptosis [42,43,44]. Notably, with regards to TNBC, Peeney and colleagues demonstrated in an orthotopic mouse model that TIMP-2 can suppress the proliferation and metastasis of TNBC tumor cells [18]. Multiple pieces of evidence have linked elevated levels of TIMP-2 with the proliferation, invasion, and/or metastasis of BC [17,45,46], as well as other cancer types such as oral cancer [47], laryngeal cancer [48], colorectal cancer [49], renal cell carcinoma [50], bladder cancer [51], and prostate cancer [52]. It is also noteworthy that other members of the TIMP family, such as TIMP-1 and TIMP-4, have been observed to exert activating influences on the proliferation of BC cells [53,54]. Moreover, high levels of serum TIMP-1 have been associated with a poor prognosis for TNBC [55].

This study has a few limitations. First, we do not have breast cancer tissues and could not compare the expression of TIMP-2 between tumor and normal tissues as well as between tumor tissues of difference stages. Second, this is a single center study, and the results need to be validated in an independent population. Finally, we only studied one gene and four SNPs. The predictive accuracy of one significant SNP is modest. Future studies should perform whole-genome SNP genotyping to identity a large panel of BC susceptibility SNPs in Taiwanese women. Then polygenic risk scores (PRS) can be developed and incorporated into risk prediction models. A personalized risk prediction model incorporating PRS can help identify women at the highest risk of developing BC, which would allow the implementation of risk stratified, targeted screening and prevention [56].

## 5. Conclusions

This is the first study to investigate the TIMP-2 SNPs and BC risk in Taiwan. We found that the homozygous variant CC genotype and the variant C allele of TIMP-2 rs8179090 SNP were associated with significantly increased BC risks in Taiwanese women. Furthermore, the association appeared to be stronger in TNBC and in younger women (≤55 years old). The other three SNPs, rs4789936, rs2009196 and rs7342880, were not associated with BC risk. This study sheds light into the etiology of BC in Taiwanese women. Rs8179090 may be incorporated into PRS and risk prediction models, which could aid in stratifying individuals for targeted breast cancer screening.

## Figures and Tables

**Table 1:** Demographics of the 1232 BC patients and the 1232 healthy controls.

Characteristic	Controls (n = 1232)			Patients (n = 1232)			*p*-Value
	n	%	Mean (SD)	n	%	Mean (SD)	
Age (years)							
<40	359	29.1%		362	29.4%		0.89 ^a^
40–55	558	45.3%		547	44.4%		
>55	315	25.6%		323	26.2%		
Age at menarche (years)			12.4 (0.7)			12.1 (0.6)	0.79 ^b^
Age at birth of first child (years)			29.4 (1.2)			29.8 (1.4)	0.63 ^b^
Age at menopause (years)			48.8 (1.8)			49.3 (2.0)	0.59 ^b^
Personal habits							
Cigarette smokers	86	7.0%		170	13.8%		0.0001 *^, a^
Alcohol drinkers	91	7.4%		162	13.1%		0.0001 *^, a^
TNBC cases							
Yes				194	15.7%		
No				1038	84.3%		
Tumor sites							
Unilateral				1198	97.2%		
Bilateral				34	2.8%		
Family history							0.6264
First degree (Mother, sister, and daughter)	46	3.7%		55	4.5%		
Second degree	5	0.4%		6	0.5%		
No history	1181	95.9%		1171	95.0%		

SD, standard deviation; ^a^ chi-square or ^b^ unpaired Student’s *t*-test; * statistically significant; TNBC, triple negative breast cancer.

**Table 2:** *TIMP-2* rs8179090 genotypes among the 1232 patients with BC and 1232 healthy controls.

Genotype	Controls		Patients		OR (95% CI)	*p*-Value ^a^
	n	%	n	%		
rs8179090						
GG	908	73.7%	841	68.3%	1.00 (Reference)	
CG	295	23.9%	317	25.7%	1.16 (0.97–1.39)	0.1249
CC	29	2.4%	74	6.0%	**2.76 (1.78–4.28)**	0.0001 *
*p*_trend_						1.00 × 10^−5^ *
*p*_HWE_						0.3886
Carrier comparison						
GG + CG	1203	97.6%	1158	94.0%	1.00 (Reference)	
CC	29	2.4%	74	6.0%	**2.65 (1.71–4.10)**	0.0001 *
GG	908	73.7%	841	68.3%	1.00 (Reference)	
CG + CC	324	26.3%	391	31.7%	**1.30 (1.09–1.55)**	0.0034 *

^a^, based on chi-square test with Yates’ correction; OR, odds ratio; CI, confidence interval; *p*_trend_, *p*-value for trend analysis; *p*_HWE_, *p*-value for Hardy–Weinberg equilibrium analysis; for significant *p*-values, they are shown as bold and a * behind.

**Table 3:** *TIMP-2* rs4789936 genotypes among the 1232 patients with BC and 1232 healthy controls.

Genotype	Controls		Patients		OR (95% CI)	*p*-Value ^a^
	n	%	n	%		
rs4789936						
CC	699	56.7%	675	54.8%	1.00 (Reference)	
CT	446	36.2%	453	36.8%	1.05 (0.89–1.24)	0.5852
TT	87	7.1%	104	8.4%	1.24 (0.91–1.68)	0.1931
*p*_trend_						0.3703
*p*_HWE_						0.1735
Carrier comparison						
CC + CT	1145	92.9%	1128	91.6%	1.00 (Reference)	
TT	87	7.1%	104	8.4%	1.21 (0.90–1.63)	0.2281
CC	699	56.7%	675	54.8%	1.00 (Reference)	
CT + TT	533	43.3%	557	45.2%	1.08 (0.92–1.27)	0.3509

^a^, based on chi-square test with Yates’ correction; OR, odds ratio; CI, confidence interval; *p*_trend_, *p*-value for trend analysis; *p*_HWE_, *p*-value for Hardy–Weinberg equilibrium analysis.

**Table 4:** *TIMP-2* rs2009196 genotypes among the 1232 patients with BC and 1232 healthy controls.

Genotype	Controls		Patients		OR (95% CI)	*p*-Value ^a^
	n	%	n	%		
rs2009196						
GG	352	28.6%	382	31.0%	1.00 (Reference)	
CG	627	50.9%	618	50.2%	0.91 (0.76–1.09)	0.3236
CC	253	20.5%	232	18.8%	0.85 (0.67–1.06)	0.1676
*p*_trend_						0.3328
*p*_HWE_						0.3904
Carrier comparison						
GG + CG	979	79.5%	1000	81.2%	1.00 (Reference)	
CC	253	20.5%	232	18.8%	0.90 (0.74–1.10)	0.3109
GG	352	28.6%	382	31.0%	1.00 (Reference)	
CG + CC	880	71.4%	850	69.0%	0.89 (0.75–1.06)	0.2014

^a^, based on chi-square test with Yates’ correction; OR, odds ratio; CI, confidence interval; *p*_trend_, *p*-value for trend analysis; *p*_HWE_, *p*-value for Hardy–Weinberg equilibrium analysis.

**Table 5:** *TIMP-2* rs7342880 genotypes among the 1232 patients with BC and 1232 healthy controls.

Genotype	Controls		Patients		OR (95% CI)	*p*-Value ^a^
	n	%	n	%		
rs7342880						
CC	869	70.5%	860	69.8%	1.00 (Reference)	
AC	334	27.1%	339	27.5%	1.03 (0.86–1.23)	0.8160
AA	29	2.4%	33	2.7%	1.15 (0.69–1.91)	0.6817
*p*_trend_						0.8428
*p*_HWE_						0.8422
Carrier comparison						
CC + AC	1203	97.6%	1199	97.3%	1.00 (Reference)	
AA	29	2.4%	33	2.7%	1.14 (0.69–1.89)	0.6996
CC	869	70.5%	860	69.8%	1.00 (Reference)	
AC + AA	363	29.5%	372	30.2%	1.04 (0.87–1.23)	0.7246

^a^, based on chi-square test with Yates’ correction; OR, odds ratio; CI, confidence interval; *p*_trend_, *p*-value for trend analysis; *p*_HWE_, *p*-value for Hardy–Weinberg equilibrium analysis.

**Table 6:** Distribution of allelic frequencies for *TIMP-2* rs8179090 among the 1232 patients with BC and 1232 healthy controls.

Allele	Controls, n	%	Patients, n	%	OR (95% CI)	*p*-Value ^a^
rs8179090						
G	2111	85.7%	1999	81.1%	1.00 (Reference)	
C	353	14.3%	465	18.9%	**1.39 (1.20–1.62)**	0.0001 *
rs4789936						
C	1844	74.8%	1803	73.2%	1.00 (Reference)	
T	620	25.2%	661	26.8%	1.09 (0.96–1.24)	0.1939
rs2009196						
G	1331	54.0%	1382	56.1%	1.00 (Reference)	
C	1133	46.0%	1082	43.9%	0.92 (0.82–1.03)	0.1522
rs7342880						
C	2072	84.1%	2059	83.6%	1.00 (Reference)	
A	392	15.9%	405	16.4%	1.04 (0.89–1.21)	0.6425

^a^, based on chi-square test with Yates’ correction; OR, odds ratio; CI, confidence interval; for significant *p*-value, it is shown as bold and a * behind.

**Table 7:** *TIMP-2* rs8179090 genotypes in BC risk after stratification by age.

Genotype	Younger (≤55), n		OR (95% CI) ^a^	aOR (95% CI) ^b^	*p*-Value	Elder (>55), n		OR (95% CI) ^a^	aOR (95% CI) ^b^	*p*-Value
	Controls	Cases				Controls	Cases			
GG	675	616	1.00 (ref)	1.00 (ref)		233	225	1.00 (ref)	1.00 (ref)	
CG	225	236	1.14 (0.93–1.42)	1.23 (0.86–1.37)	0.2193	70	81	1.20 (0.83–1.73)	1.32 (0.89–1.82)	0.3851
CC	17	57	**3.67 (2.11–6.38)**	**3.72 (2.05–6.45)**	0.0001 *	12	17	1.47 (0.69–3.14)	1.51 (0.75–3.31)	0.4236
Total	917	909				315	323			
*p*_trend_					4.68 × 10^−6^ *					0.4268

^a^, by multivariate logistic regression analysis; ^b^, by multivariate logistic regression analysis after adjusting for gender, smoking and alcohol drinking status; *p*_trend_, *p*-value for rend analysis; *, statistically significant; CI, confidence interval; aOR, adjusted odds ratio; * and bolded, statistically significant.

**Table 8:** Association of *TIMP-2* rs8179090 genotypes with BC risk stratified with TNBC, non-TNBC, or healthy controls.

Genotype	Control	Non-TNBC	OR, 95%CI	*p*-Value ^a^	TNBC	OR, 95%CI	*p*-Value ^a^
GG	908	715	1.00 (Ref)		126	1.00 (Ref)	
CG	295	263	1.13 (0.95–1.37)	0.2255	54	1.32 (0.93–1.86)	0.1373
CC	29	60	**2.63 (1.67–4.14)**	0.0001 *	14	**3.48 (1.79–6.76)**	0.0003 *
Total	1232	1038			194		
*p*_trend_				6.97 × 10^−5^ *			0.0003 *

^a^, based on chi-square test without Yates’ correction; OR, odds ratio; CI, confidence interval, TNBC, triple negative breast cancer; *p*_trend_, *p*-value for trend analysis; * and bolded, statistically significant.

## Data Availability

The genotyping results and clinical data supporting the findings of this study are available from the corresponding authors upon reasonable requests via email at artbau2@gmail.com.
